# Comparing the Effects of Different Body Armor Systems on the Occupational Performance of Police Officers

**DOI:** 10.3390/ijerph15050893

**Published:** 2018-05-01

**Authors:** Ben Schram, Robin Orr, Rodney Pope, Ben Hinton, Geoff Norris

**Affiliations:** 1 Tactical Research Unit, Bond University, Robina, QLD 4226, Australia; bschram@bond.edu.au (B.S.); rpope@csu.edu.au (R.P.); 2 School of Community Health, Charles Sturt University, Albury, NSW 2640, Australia; 3 New South Wales Police—Health and Fitness Unit, Sydney, NSW 2150, Australia; hint1ben@police.nsw.gov.au; 4 New South Wales Police—Operational Safety and Skills Command, Sydney, NSW 2150, Australia; norr1geo@police.nsw.gov.au

**Keywords:** light armor, personal protective equipment, load, law enforcement, occupational tasks

## Abstract

Policing duties may inherently be dangerous due to stab, blunt trauma and ballistic threats. The addition of individual light armor vests (ILAVs) has been suggested as a means to protect officers. However, the addition of the extra load of the ILAV may affect officer ability to conduct occupational tasks. The purpose of this study was to determine if wearing any of three different ILAVs made by different companies with their preferred materials and designs (ILAV A, 4.68 percent body weight, ILAV B, 4.05 percent body weight, & ILAV C, 3.71 percent body weight) affected occupational task performance when compared to that in normal station wear. A prospective, within-subjects repeated measures design was employed, using a counterbalanced randomization in which each ILAV was worn for an entire day while officers completed a variety of occupationally relevant tasks. These tasks included a victim drag, car exit and 5-meter sprint, step down and marksmanship task. To compare the effects of the ILAVs on these tasks, a multivariate repeated measures analysis of variance (ANOVA) was conducted, with post hoc pairwise comparisons using a Bonferroni adjustment. Results showed that performance in each task did not vary between any of the ILAV or normal station wear conditions. There was less variability in the marksmanship task with ILAV B, however. The results suggest that none of the ILAVs used in this study were heavy enough to significantly affect task performance in the assessed tasks when compared to wearing normal station wear.

## 1. Introduction

In an age of increasing threats, police forces are more commonly utilizing Individual Light Armor Vests (ILAVs) for protection of their officers from occupational risks such as stabbing, blunt trauma, and gunshot wounds [1,2]. The ILAVs used in police forces tend to be lighter (~2.7–3.8 kg) than military armor; however, they still contribute to an overall extra load on an officer [3]. The extra load of an ILAV is added to the equipment employed by officers, which may include items such as a communication system, weaponry, handcuffs and torches [1]. The weight of this load can range from 3 kg to 15 kg [3] and has been shown to decrease occupation-specific performance in police officers, for example, increasing the time required to complete a 5-meter sprint, vehicle exit task, ground mobility and grapple task [1]. Ideally, ILAV should provide optimum protection for officers without hindering performance by restricting movements or slowing their pursuit of persons of interest. 

There have been numerous investigations into the negative effects of carrying excessive loads in military populations [4,5,6,7,8], with recommendations that no more than 30 percent body weight be carried to avoid detrimentally affecting performance [8]. In military and specialist law enforcement situations, the addition of load, inclusive of body armor, has been found to decrease mobility [5], increase time required to move between cover and negotiate obstacles [9] and slow completion of shooting, vaulting and crawling tasks [7]. With respect to operating weapon systems, increases in load have also been shown to increase the time taken to engage a target [10] and decrease accuracy of throwing a grenade [8]. However, there have been conflicting results regarding the impacts of loads on both accuracy and precision in marksmanship tasks, with some authors hypothesizing that these may be more affected by fatigue than the load directly [8]. Other authors have found trends toward improved marksmanship with ballistic vests, possibly due to a stabilization effect on the shoulders [11]. Despite many investigations into the effects of additional load in military populations, there is minimal evidence to indicate the effects of body armor, specifically. Furthermore, investigations have typically focused on comparing one type of body armor against other load conditions (for example no-load and tactical load [11]), as opposed to comparing different types of body armor.

Prior to any decisions being made on the large-scale recommendation and utilization of ILAVs by police forces, a greater understanding is required of both the effects of the added ILAV loads, and any potential differences in occupational performance resulting from the wearing of specific types of ILAV. Therefore, the purpose of this study was to compare the effects of wearing each of three different ILAVs and of normal station wear on the performance of occupational tasks in police officers. 

## 2. Materials and Methods 

To ensure the sample selected was representative of the general state police population, two small-, medium- and larger-stature male and female serving police officers were initially recruited. This process was designed to ensure the translatability of this research to the entire police force and to provide understanding of the effects of the ILAVs when applied to a range of body sizes from both sexes. Each officer was initially briefed, and all expressed a willingness to participate, providing written informed consent. One female officer was removed at this stage from the study due to a medical concern, and the sample was reduced to 11 officers, comprising five females (mean ± SD age = 27 ± 3 years; weight = 68 ± 18 kg; height = 164 ± 7cm; months of service = 78 ± 12 months) and six males (mean ± SD age = 40 ± 8 years; weight = 83 ± 20 kg; height = 177 ± 9.0 cm; months of service = 92 ± 9 months).

A prospective, within-subjects, repeated measures, study design was employed, using a counterbalanced randomization protocol by which to allocate one of four load condition types, being ILAV types A, B and C and “normal” (N) station wear, where officers wore their own station wear. Each officer served as their own control and, regardless of which load condition they were randomly allocated on the first day of data collection, they progressed to the next load condition type in the following predetermined order: ILAV A, ILAV B, ILAV C, N and from N to ILAV A. Officers were required to wear each of their allocated load conditions for an entire day over the 4 days of the study period. This procedure was aimed at reducing any learning effect and the effect of any external factors such as variable weather conditions, which may have occurred over the duration of the study. 

Data collection for the study took place at a state police college in 2016 over the 4-day period. The ambient temperature and relative humidity across the testing times ranged from 12 to 24 °C and from 36 to 93 percent, respectively, giving a heat stress index varying between 11.4 and 22.6 °C during the testing period. This study was approved by the Bond University Human Research Ethics Committee (protocol number 15803), and all participants formally consented to participate. Departmental approvals for the conduct of the study and release of this paper were also obtained. 

### 2.1. Outcome Measures

The same testing procedure was used each day to minimize diurnal variation. As this data capture was part of a larger study, the measures relevant to this study are displayed in Table 1. Each activity is described in more detail below. 

### 2.2. 10 m Victim Drag

**Figure 1:**
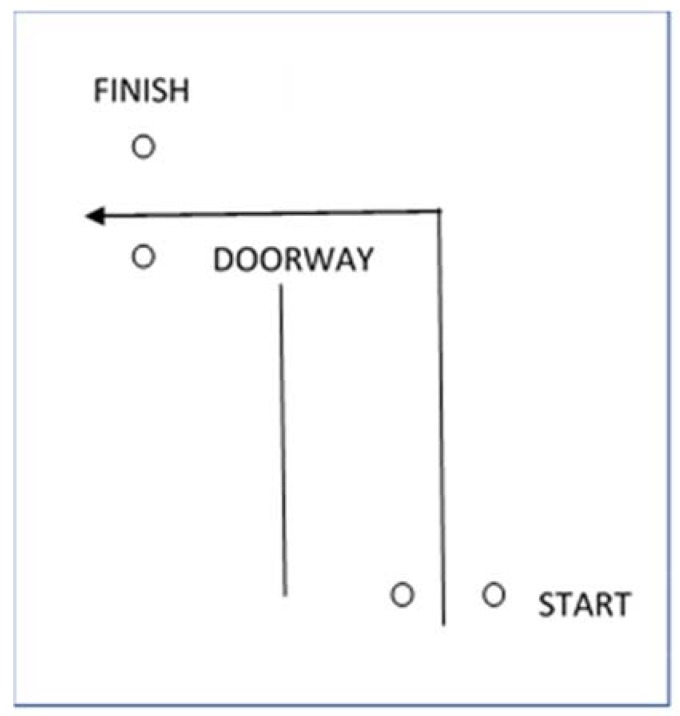
10 m Victim drag course.

A victim drag scenario was set up utilizing a 60 kg mannequin fitted with a 10 kg weighted vest. The recovery course required the officer to drag the mannequin 6 m directly backward, then negotiate a 90-degree left hand turn through a doorway, before dragging the victim another 4 m to the end of the track (Figure 1). This configuration was designed to mimic retrieving a victim from an exposed area and then dragging them back and behind cover. All times were recorded using a light-beam SMARTSPEED timing gate system (Fusion Sport, Queensland: Australia). The distances officers covered when completing the recovery course were measured using a digital mini-measuring wheel (Senshin Industry Co., Ltd., Osaka, Japan). Officers were allowed an initial practice run at approximately 80 percent of their maximum ability to familiarize themselves with the scenario and for a warm up on each day of testing. Time was recorded to the nearest second.

### 2.3. Car Exit and 5 m Sprint to Cover

**Figure 2:**
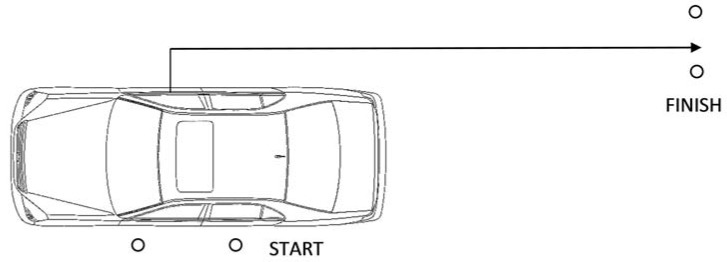
Car exit and 5 m sprint layout.

A standard police patrol car (General Motors Holden Commodore SS Sedan) was parked on the side of a track for a vehicle exit scenario. Officers were seated in the driver’s seat of the vehicle without a seatbelt on and with both hands on the steering wheel. A verbal command was given by the researcher to start the scenario, whilst their hand was used to break the light beam of the SMARTSPEED timing gate system (Fusion Sport, Queensland: Australia) and so to start the timer. The officer exited the driver’s side of the vehicle and ran 5 m rearward, with the distance measured using a digital mini-measuring wheel (Senshin Industry Co., Ltd., Osaka, Japan), through the corresponding timing gate as quickly as possible (Figure 2). Officers were given only one opportunity to complete this scenario. Time was recorded in seconds. 

### 2.4. Curb Step Down

The curb step down task required the officer to step off a 20 cm step onto the Fitness Technology Force Platform to determine the peak ground reaction force. The officers were given a verbal command to ‘go’ and were required to step off the step onto the platform and then off the platform in a natural gait pattern. The aim of this task was to gain an appreciation of the loads placed through the officer’s body when simulating a step off a street curb side.

### 2.5. Marksmanship

The marksmanship task involved officers engaging a target (human silhouette live fire target with four scoring zones) with a Glock model 22 pistol, firing 26 rounds in total, and was scored over three separate sequences. These three sequences were: Point/proximity shooting (nine rounds); Immediate distance/kneeling (five rounds); and Transition drills/reloading (12 rounds). Each sequence assessed a single or related multiple skill set and these were deemed mandatory and necessary skills for the operational policing environment. To score points for assessable hits, one point was awarded when the whole of the round impression/cut was wholly within the human silhouette. However, if the round cut a line and any portion of the ‘cut’ lay within the next zone, the shooter was awarded the higher points. The maximum score was 104 points. For the duration of the marksmanship task, the volunteer officers were under the authority of the host Police Force and their qualified range instructors. 

### 2.6. Statistical Analyses

All recorded data, except for data relating to ambient weather conditions, were entered into a data spreadsheet in SPSS version 23 (IBM 2015, SPSS Inc., Chicago, IL, USA). Initial descriptive analyses were then conducted to provide counts, means, standard deviations and ranges for the included variables, as relevant depending on levels of measurement. These descriptive statistics were derived for each sex and for each body armor type where relevant, as well as for the entire sample. 

Following these descriptive analyses, multivariate repeated measures analyses of variance (ANOVA) were conducted to examine the effects of body armor type on key performance measures, with post hoc pairwise comparisons using a Bonferroni adjustment. Results were graphed where this approach provided useful visualization of key differences. The overall level of significance was set a priori at 0.05.

Data relating to ambient weather conditions were analyzed descriptively to determine the range of ambient temperatures, levels of relative humidity and range of heat stress index scores observed during data collection times on the four data collection days. These have been reported in the Methods section of this report.

## 3. Results

An overview of the weight of each ILAV can be found in Table 2, with each ILAV being from different companies using their preferred materials and design. The mean weights varied between body armor types by 0.3 to 0.9 kg. Maximum weights (reflecting the largest ILAV sizes) varied between body armor types by 0.7 to 1.5 kg, indicating differences of possible practical or operational significance. There were significant differences between the mean weights of all three ILAV types (*p* < 0.05 for all on Bonferroni post-hoc tests; Table 2). The differences in ILAV weights were mitigated to some degree when officers were fully equipped with daily work equipment (e.g., handcuffs, radio, etc.) (Table 2); however, they were all still significantly heavier than the loads involved in wearing normal station wear alone (*p* < 0.002 for all on Bonferroni post-hoc tests).

The results for the victim drag, car exit and step-down task are provided in Table 3. The quickest time was recorded for ILAV B, and was 0.27 s (4.94 percent) quicker than the slowest, for ILAV A. However, there were no significant differences between any of the ILAV and N conditions in times to complete the victim drag task (F(3,30) = 0.753, *p* = 0.529). 

The quickest time was recorded with ILAV C, and was 0.09 s (2.6 percent) faster than for ILAV A. Again, however, there were no significant differences between any of the ILAV and N conditions in time to complete the car exit and 5m sprint to cover (F(3,30) = 0.390, *p* = 0.761). 

The highest peak force occurring during the step-down task was seen for ILAV B, which was 7.8 percent higher than the lowest peak force, which was associated with ILAV C. There were, however, no significant differences in the peak force readings from the curb step-down task between any of the ILAV and N conditions (F(1.607,16.071) = 0.865, *p* = 0.417). 

**Figure 3:**
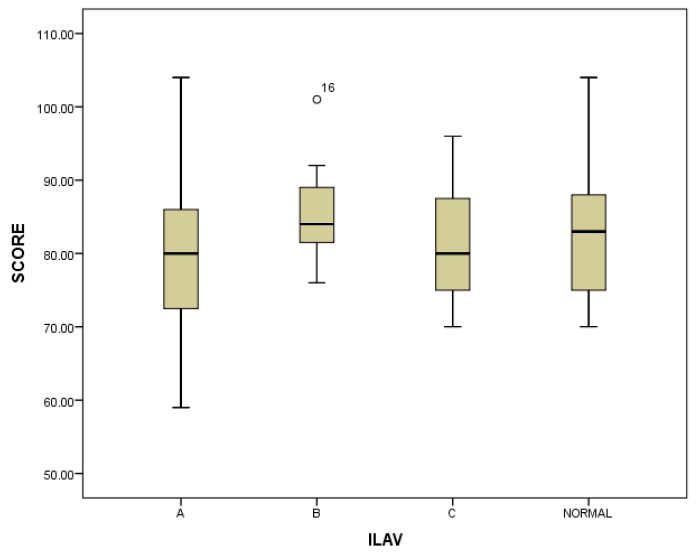
Marksmanship scores across load conditions.

The results of the marksmanship task are shown in Figure 3. Overall, the differences between average marksmanship scores achieved whilst wearing the different ILAV or N conditions were small and did not reach statistical significance (average marksmanship scores: ILAV A 80.7, ILAV B 85.6, ILAV C 81.5, normal station wear 83.8; F(3,30) = 2.124, *p* = 0.118). Considering this, as shown in Figure 1, below, wearing ILAV B was associated with the least variability in shoot scores.

## 4. Discussion

The purpose of this study was to determine the effects various ILAV systems had on the occupational task performance of state police officers. In contrast to the effects on military tasks, the addition of the ILAV to normal station wear did not affect officer performance in any of the occupational tasks observed in this study. It is suggested that the weights of the ILAVs and associated equipment loads used by the officers in the current study were not heavy enough to significantly affect performance of the occupational tasks assessed, in contrast to wearing heavier external loads, for example over 20 kg for specialist police [12] and over 40 kg in military personnel [5,13]. It should be noted, however, that these lighter ILAVs may sacrifice a degree of passive protection from heavier armor in exchange for active protection in the form of increased movement speed.

Performance in the victim drag task in this study was not significantly affected by wearing any of the individual ILAV or N load conditions. In contrast, in another study, a variation of this same task, a 10 m victim drag of a mannequin, was shown to be performed significantly more slowly when wearing body armor and carrying additional load of over 20 kg in weight [12]. The lighter vests used in the current study may in fact be light enough to provide a degree of protection, while not hindering occupational task performance. When considering the lack of significant differences in performance of a car exit and sprint observed across the different ILAV or N conditions, the results of the current study are in contrast to the findings by Dempsey [1]. In their study, Dempsey [1] asked participants, while wearing stab-resistant body armor in conjunction with appointments (7.65 ± 0.73 kg), to exit a patrol car and sprint 2.85 m. The time to completion was significantly (*p* < 0.001) longer in the loaded condition (+0.28 s). It should be noted, however, that in the studies by Carlton et al. [12] and Dempsey [1], the differences in load trial conditions were notably larger. In the study by Carlton et al. [12], the difference between the unloaded and loaded condition was approximately 17 kg. Likewise, in the study by Dempsey [1], the difference in weights between conditions was approximately 7.65 kg. The smaller differences in weights between load conditions in the current study (2.90–5.50 kg) provide a potential reason for the differences between the findings of this study and those of Carlton et al. [12] and Dempsey [1]. 

The step-down task did not differ in peak force readings, regardless of load condition. Previous research on police officers has found a drop landing from a 0.75 m platform while wearing body armor and accessories (7.65 ± 0.73 kg) led to significantly greater peak ground reaction forces upon landing when compared to those observed in an unloaded condition [3]. Both the heavier weights and greater height from which officers dropped in this previous research likely contributed to the difference in results between that study and the current study. 

The results from the marksmanship task showed less variability in shoot scores when wearing ILAV B, but no significant difference overall between any of the ILAV and N conditions. Previous research has shown that marksmanship accuracy can be affected after a load carriage activity for 45 min in soldiers (30.5 ± 1.5 kg) [14] and after a 20 km road march carrying load (46 kg) [15]. This decrease in performance may be due to fatigue, rather than the extra load having a direct effect on marksmanship [8]—it is known that carrying heavy loads increases metabolic cost [16], which may increase fatigue [17]. The marksmanship task in this study was performed without any fatigue and without strict time constraints, and this may have affected the findings. While the majority of other marksmanship studies have used rifles, Carbone et al. [11] studied the effects of load on marksmanship using handguns. No real difference was found between load conditions in the study of Carbone et al. [11], which is in line with our findings. Any impact of elevations in heart rate or respiratory rate associated with fatigue or exercise may affect primary weapons that are in contact with the thoracic wall and shoulder but not so much small arms such as the handguns used in this study and that of Carbone et al. [11], as they are at arm’s length. Marksmanship is also measured and reported in a number of different ways by authors. These include accuracy, indicated by number of hits on target [10] and their distances from the center of mass of a target [8,10], and precision, expressed as hit group size [8,10]. Different methods of reporting marksmanship may lead to dissimilar results. Overall, this study found that there were no significant differences between any of the ILAV or N conditions in occupational performance in any of the observed tasks. As mentioned previously, this may be due to the relatively lighter weights of the ILAVs investigated in this study and the fact that that the relative loads of the ILAVs were small when compared to the overall load which an officer carried (ILAV A: 4.68 percent body weight, ILAV B: 4.05 percent body weight & ILAV C 3.71 percent body weight). 

It should be acknowledged that these results are reflective of only the observed tasks, and did not extend to other factors that may be important to policing. Despite no observed significant effects of the ILAVs on officer performance of any of the occupational tasks investigated in this study, other considerations which may affect the choice of one ILAV over another may include the subjective opinion of officers, and the effects of the ILAV on mobility and balance and range of motion. Due to the time constraints on this study, there was also no capacity to examine the chronic impacts of sustained ILAV loads on the musculoskeletal system of the officers, and prospective studies designed to examine this issue would be of great value given that officers are increasingly wearing ILAV in their day-to-day duties and for increasing numbers of hours—in some cases constantly during working hours. 

## 5. Conclusions

The results from this study suggest that while ILAVs may be significantly heavier than normal station wear, they may not have a notable impact on police officer performance of victim drag tasks, vehicle exits and marksmanship tasks. In addition, musculoskeletal loadings may not be significantly greater when stepping off a low curb when wearing ILAVs than when wearing normal station wear. Police forces can be confident that the addition of load through adopting ILAVs of similar weights to those investigated in the current study will not significantly affect officer performance of a range of representative occupational tasks. However, a caution is noted in that the chronic musculoskeletal effects of sustained increases in carried loads, even slight increases, require further investigation. 

## Figures and Tables

**Table 1:** The daily sequence of events.

Time	Measure
08:00	Equipment preparation
09:00	Victim Drag
11:00	Car exit with 5 m sprint
13:00	Step Down Task
14:45	Marksmanship

**Table 2:** Mean ± SD and ranges for each type of ILAV and stationwear (N) in all configurations.

ILAV Type (A-C) & Normal Station Wear (N)	ILAV Weight (kg)	Duty Load Complete (kg)	Total Load Including Officer Weight (kg)
A	4.12 ± 0.65 *^,^**	11.53 ± 0.77 ^‡^	88.03 ± 20.49
B	3.54 ± 0.70 **	11.01 ± 1.01 ^‡^	87.51 ± 20.60
C	3.24 ± 0.48 *	10.77 ± 1.16 ^‡^	87.27 ± 20.66
N	NA (0)	8.69 ± 0.68	85.19 ± 20.24

Significantly different (*p* < 0.05) from * ILAV B ** ILAV C: ^‡^ Significantly different (*p* < 0.001) from normal station wear.

**Table 3:** Task results. Results expressed as mean ± SD.

	Victim Drag	Car Exit	Step Down
Condition	Time (s)	Time (s)	Peak Force (N)
ILAV A	5.74 ± 0.28	3.49 ± 0.94	1734 ± 382
ILAV B	5.47 ± 0.23	3.41 ± 0.87	1797 ± 463
ILAV C	5.50 ± 0.38	3.40 ± 1.06	1667 ± 449
N	5.56 ± 0.43	3.41 ± 0.85	1682 ± 383
